# Motion and anatomy dual aware lung ventilation imaging by integrating Jacobian map and average CT image using dual path fusion network

**DOI:** 10.1002/mp.17466

**Published:** 2024-10-21

**Authors:** Pei Ma, Zhi Chen, Yu‐Hua Huang, Mayang Zhao, Wen Li, Haojiang Li, Di Cao, Yi‐Quan Jiang, Ta Zhou, Jing Cai, Ge Ren

**Affiliations:** ^1^ Department of Health Technology and Informatics The Hong Kong Polytechnic University Kowloon Hong Kong SAR Hong Kong; ^2^ Department of Radiology State Key Laboratory of Oncology in South China Collaborative Innovation Centre for Cancer Medicine Guangdong Key Laboratory of Nasopharyngeal Carcinoma Diagnosis and Therapy Sun Yat‐sen University Cancer Centre Guangzhou People's Republic of China; ^3^ Department of Minimally Invasive Interventional Therapy State Key Laboratory of Oncology in South China Guangdong Provincial Clinical Research Center for Cancer Sun Yat‐sen University Cancer Center Guangzhou People's Republic of China; ^4^ The Hong Kong Polytechnic University Shenzhen Research Institute Shenzhen Guangdong Province People's Republic of China

**Keywords:** CT ventilation imaging, deep learning, functional lung avoidance radiotherapy, Jacobian map

## Abstract

**Background:**

Deep learning‐based computed tomography (CT) ventilation imaging (CTVI) is a promising technique for guiding functional lung avoidance radiotherapy (FLART). However, conventional approaches, which rely on anatomical CT data, may overlook important ventilation features due to the lack of motion data integration.

**Purpose:**

This study aims to develop a novel dual‐aware CTVI method that integrates both anatomical information from CT images and motional information from Jacobian maps to generate more accurate ventilation images for FLART.

**Methods:**

A dataset of 66 patients with four‐dimensional CT (4DCT) images and reference ventilation images (RefVI) was utilized to develop the dual‐path fusion network (DPFN) for synthesizing ventilation images (CTVI_Dual_). The DPFN model was specifically designed to integrate motion data from 4DCT‐generated Jacobian maps with anatomical data from average 4DCT images. The DPFN utilized two specialized feature extraction pathways, along with encoders and decoders, designed to handle both 3D average CT images and Jacobian map data. This dual‐processing approach enabled the comprehensive extraction of lung ventilation‐related features. The performance of DPFN was assessed by comparing CTVI_Dual_ to RefVI using various metrics, including Spearman's correlation coefficients (*R*), Dice similarity coefficients of high‐functional region (DSC_h_), and low‐functional region (DSC_l_). Additionally, CTVI_Dual_ was benchmarked against other CTVI methods, including a dual‐phase CT‐based deep learning method (CTVI_DLCT_), a radiomics‐based method (CTVI_FM_), a super voxel‐based method (CTVI_SVD_), a Unet‐based method (CTVI_Unet_), and two deformable registration‐based methods (CTVI_Jac_ and CTVI_HU_).

**Results:**

In the test group, the mean *R* between CTVI_Dual_ and RefVI was 0.70, significantly outperforming CTVI_DLCT_ (0.68), CTVI_FM_ (0.58), CTVI_SVD_ (0.62), and CTVI_Unet_ (0.66), with *p* < 0.05. Furthermore, the DSC_h_ and DSC_l_ values of CTVI_Dual_ were 0.64 and 0.80, respectively, outperforming CTVI_SVD_ (0.63; 0.73) and CTVI_Unet_ (0.62; 0.77). The performance of CTVI_Dual_ was also significantly better than that of CTVI_Jac_ and CTVI_HU_.

**Conclusions:**

A novel dual‐aware CTVI model that integrates anatomical and motion information was developed to synthesize lung ventilation images. It was shown that the accuracy of lung ventilation estimation could be significantly enhanced by incorporating motional information, particularly in patients with tumor‐induced blockages. This approach has the potential to improve the accuracy of CTVI, enabling more effective FLART.

## 1 INTRODUCTION

Lung cancer is the leading cause of cancer‐related mortality globally.^1^ Radiotherapy (RT) plays a crucial role in the management of lung cancer, serving as either a primary or complementary therapy. This approach is particularly valuable for patients who are not candidates for surgical intervention or have advanced‐stage cancers.^2^ In the era of precision medicine, functional lung avoidance radiotherapy (FLART) has been proposed as a novel RT approach to improve treatment accuracy for lung cancer. It aims to minimize radiation exposure to high‐functional lung regions, thereby reducing the risk of RT‐related lung toxicity and improving treatment outcomes.^3^, ^4^, ^5^

To implement FLART, functional images are essential for designing the treatment plan. Nowadays, various pulmonary function imaging methods, such as single‐photon emission computed tomography (SPECT) perfusion imaging with technetium‐99 m (Tc‐99 m)^6^ and positron emission tomography (PET) ventilation imaging with gallium‐68 (Ga‐68),^7^ have been explored to visualize blood supply and airflow, respectively. However, the spatial resolution of these modalities might not be sufficient for high‐quality imaging in this application, thus reducing their usefulness in treatment planning. In addition, the free‐breathing acquisition modality of SPECT/PET can lead to respiratory motion‐related blurring.^8^ Hyperpolarized noble gas magnetic resonance imaging (MRI)^9^, ^10^, ^11^, ^12^ provides a non‐invasive, radiation‐free method for assessing lung function, but is difficult to implement clinically due to the limited availability of the special gas. In general, all these methods require additional scan(s) that are typically not of interest to FLART.

An alternative method is to estimate the ventilation images from non‐contrast respiratory‐correlated CT datasets, named as CT ventilation imaging (CTVI) method. Conventional CTVI methods primarily rely on four‐dimensional CT (4DCT) and deformable image registration (DIR) to analyze motion‐related changes in lung volume (Jacobian‐based CTVI [CTVI_Jac_])^13^ or lung density (Hounsfield unit [HU]‐based CTVI [CTVI_HU_]).^14^ The HU‐based CTVI method assumes lung tissue density correlates with ventilation ability. When air volume increases during inhalation, overall lung tissue density is reduced. DIR aligns lung voxels across different breathing phases for measuring HU value changes, which are then utilized to estimate ventilation map. The Jacobian‐based method focuses on lung tissue deformation, using the DIR‐generated deformation field to calculate the Jacobian determinant, which measures local volumetric expansion or contraction. The Jacobian‐based method provides a direct measure of lung tissue expansion and contraction. While the HU‐based method is less sensitive to DIR errors, the general accuracy of these two methods depends on the DIR accuracy. In addition to the mentioned techniques, conventional CTVI methods also incorporate radiomics and supervoxel techniques to enhance the precision of lung ventilation estimation.^15^, ^16^, ^17^, ^18^

With the rapid development of deep learning technology, several deep learning‐based CTVI methods have achieved high accuracy and outperformed conventional CTVI methods in generating ventilation images.^19^, ^20^, ^21^, ^22^ These deep learning models focus on direct feature extraction from the anatomical information provided by different inputs, such as peak‐inhale/peak‐exhale CT images (CT_in_/CT_ex_), 4DCT images, and three‐dimensional CT (3DCT) images.^20^, ^21^, ^22^ However, lung ventilation is influenced not only by lung texture changes but also by other conditions. Yuan et al. reported that tumor‐related airway blockages can lead to temporary loss of function in the affected lung regions.^23^ Most existing deep learning models overlook this blockage‐induced ventilation modality change, misclassifying them as high‐functional regions. To overcome this limitation, this study seeks to incorporate a more comprehensive range of factors that influence ventilation function changes, including anatomical information from CT images and motion data derived from the DIR. By integrating these diverse data sources, we aim to develop and evaluate a more accurate deep learning‐based approach for CTVI.

## 2 METHODS

### 2.1 Patient cohort

A total of 66 patients with reference ventilation images (RefVI) from two sources were included for the model development (Table 1). The first dataset comprised 20 patients from The Cancer Imaging Archive (TCIA), which provided 4DCT scans, Galligas PET scans, and 19 CT scans (one patient, CT‐PET‐VI‐07, was missing the CT scan).^24^ The second dataset comprised 46 patients from the Ventilation And Medical Pulmonary Image Registration Evaluation (VAMPIRE) challenge.^25^ In the second dataset, 21 patients had 4DCT images, time‐averaged 4DCT images, diethylenetriamine pentaacetate acid SPECT (DTPA‐SPECT) ventilation, and corresponding lung masks.^26^ The remaining 25 patients from the Peter MacCallum Cancer Center had Galligas 4DPET/CT and 4DCT images.^27^, ^28^, ^29^ All RefVI were rigidly registered using 4DCT in the respective original datasets. Figure S1 shows the selected images from the three datasets.

**TABLE 1:** Summary of functional lung imaging data included in this study.

Sources	Name	Galligas 4DPET/CT	DTPA‐SPECT	Galligas PET/CT
	Institution	Peter MacCallum Cancer Centre	Stanford University	Royal North Shore Hospital
	Patient type	Lung cancer	Lung cancer	Lung cancer
	Number	25	21	20
	Task	Training/validation	Test	Training/validation
4DCT scans	Scanner type	4DPET/CT	4DCT	4DCT
	Breathing condition	Free‐breathing	Free‐breathing	Free‐breathing
	Acquisition mode	Cine	Cine or helical	Helical
	Phase bins	5	10	10
	Slice thickness	5.0 mm	2–3 mm	1.7 mm
	In‐plane resolution	1:07 × 1:07 mm^2^	0:97 × 0:97 mm^2^	0.96 × 0.96 mm^2^
	Tube voltage/current:	140 kVp/10 mA	120 kVp/100 mAs /slice	120 kVp/80–200 mA
RefVI scans	Scanner type	4DPET/CT	SPECT/CT	PET/CT
	Radiotracer	68Ga	99mTc	68Ga
	Axial coverage	Whole lung	Whole lung	Whole lung
	Slice thickness	3.27 mm	8 mm	2.2 mm
	In‐plane resolution	2.87 × 2.87 mm^2^	8 × 8 mm^2^	2.04 × 2.04 mm^2^
	Scanner	GE Discovery 690 PET/CT	GE Infinia Hawkeye SPECT/CT	Siemens Biograph mCT S/64 PET/CT

Abbreviations: 4DCT, four‐dimensional computed tomography; DTPA, diethylenetriamine pentaacetate acid; PET, positron emission tomography; RefVI, reference ventilation images; SPECT, single photon emission computed tomography.

### 2.2 Image preprocessing

To minimize computational cost, only the smallest bounding box containing the lungs was cropped from the CT scans. A median filter was applied to the ventilation images as a smoothing prior in order to mitigate noise. Automatic lung‐mask generation and Jacobian map calculation^30^ were respectively evaluated using a well‐trained Unet (R231) model^31^ and CT imaging (CT_in_ and CT_ex_). In addition, DIR between CT_in_ and CT_ex_ was performed using the pTVreg registration method.^32^ Subsequently, the Jacobian map was calculated using Equation (1):

(1) $$\begin{array}{cc} & \mathrm{Jacobian}\;\left(x,y,z\right)\\ & =\left|\begin{array}{ccc} 1+\frac{\partial u_{x}\left(x,y,z\right)}{\partial x} & \;\frac{\partial u_{x}\left(x,y,z\right)}{\partial y} & \;\frac{\partial u_{x}\left(x,y,z\right)}{\partial z}\\ \;\frac{\partial u_{y}\left(x,y,z\right)}{\partial x} & 1+\frac{\partial u_{y}\left(x,y,z\right)}{\partial y} & \;\frac{\partial u_{y}\left(x,y,z\right)}{\partial z}\\ \frac{\partial u_{z}\left(x,y,z\right)}{\partial x} & \frac{\partial u_{z}\left(x,y,z\right)}{\partial y} & 1+\frac{\partial u_{z}\left(x,y,z\right)}{\partial z} \end{array}\right|-1\; \end{array}$$

where function u(x,y,z) is the voxel displacement field, $u_{x}\left(x,y,z\right)$ is the *x* component of u(x,y,z), $u_{y}\left(x,y,z\right)$ is the *y* component of u(x,y,z), and $u_{z}\left(x,y,z\right)$ is the *z* component of u(x,y,z).

The cropped CT scans and Jacobian maps were resampled to a size of 60 × 60 × 60. To normalize the CT values for deep learning model training, the values within the range of [‐1000, 0] were rescaled to [0, 1] using a proportional linear transformation method. Min‐max normalization was applied to the Jacobian image values. To mitigate the effect of hotspots in RefVI, the 90th percentile value was used as the threshold for identifying hyper‐functioning lung regions.^33^ Values of RefVI exceeding this threshold were reset to the threshold value and subsequently normalized to the range [0, 1].

### 2.3 Architecture of neural network

**FIGURE 1:**
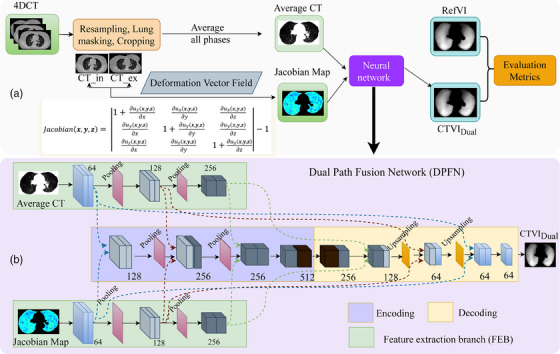
The overall flowchart (a) and the DPFN design (b). DPFN, dual path fusion network.

To leverage the anatomical information from average CT images and motional information from Jacobian maps for ventilation imaging (CTVI_Dual_), a 3D dual‐path fusion network (DPFN) was developed based on the architecture of our previously developed multimodality‐guided synergistic neural network (MMgSN‐Net).^34^ The original MMgSN‐Net was designed to extract information from 2D images, which limited its ability to capture the depth information inherent in CT images. The proposed method is based on two feature extraction branches (FEBs), an encoder, and a decoder to effectively integrate the 3D data from the average CT images and Jacobian maps (Figure 1).

#### 2.3.1 Feature extraction branches

The two FEBs were specifically designed to extract features from the input CT images and Jacobian maps, respectively. These FEB modules in the network focused on different aspects due to the inherent differences in the data. The FEB of CT image focuses on extracting anatomical information, whereas the Jacobian FEB specifically targets motional information. Each branch consists of three convolutional blocks with a kernel size of 3 × 3 × 3 and two pooling layers. To ensure feature normalization, a batch normalization layer was applied after each convolutional block.

#### 2.3.2 Encoder

To integrate the information extracted from each branch, feature maps with 64, 128, and 256 dimensions were generated from the first, second, and third convolutional blocks, respectively. The features were then continuously fused using a fusion layer encoder, which comprised four convolutional blocks with channel sizes of 128, 256, 256, and 512, along with two pooling layers. The fusion process employed a combination method^35^ that included voxel‐wise summation, products, and maximization. These operations effectively integrated different features to obtain useful complementary information for the synthesis of ventilation images.

#### 2.3.3 Decoder

The network employed a decoder module to upsample and merge the feature maps extracted by the encoder, gradually restoring image details and spatial information. The decoder primarily consisted of two upsampling layers and three convolutional layers with channel sizes of 256, 128, and 64, respectively. Trilinear interpolation was employed to upsample the features to match the size of the feature map. Subsequently, this upsampled feature map was convolved with features from the corresponding layer of the feature extraction branches to restore the image details. Finally, a reconstructed ventilation image was generated in the final layer of the network structure.

### 2.4 Training and implementation details

To prevent overfitting during training, several data augmentation techniques were applied, including flipping in all three directions with a 50% probability, left‐right random rotation angles within (‐15, 15), random cropping from the borders within 10% of the data size, and elastic transformation within 25% of the data size using the “Volumentations 3D”.^36^ To evaluate the effect of data augmentation on the prevention of overfitting, a comparative analysis was conducted by training a model without augmentation.

The network was trained for 800 epochs via a decay‐based learning strategy using the Adam optimizer, with an initial learning rate of 0.0001 and a batch size of 1. The decay factor of the learning rate is determined using the current epoch. If the current epoch surpasses the starting decay epoch, the decay factor can be computed by subtracting the ratio from 1.0; the ratio can be obtained by dividing the difference between the current and starting decay epochs by the difference between the total and starting decay epochs, thereby initiating learning rate decay. In addition, the mean absolute error was considered as the loss function during training.

The implementation was performed using PyTorch 1.8 on a Windows‐based computer with Intel Core i9‐12900F CPU (5.1 GHz), NVIDIA RTX 3090 GPU (24GB memory), and 64GB RAM.

### 2.5 Evaluation of CTVI_Dual_

The performance of the model was assessed using a three‐fold cross‐validation scheme. Of the 66 patients, 45 with PET images as RefVI were randomly divided into three folds. In each iteration, two folds were used for training the model, and the remaining fold was used for validation. Additionally, the data from the remaining 21 participants with SPECT RefVI were reserved as an external test dataset to assess the model's generalization ability.

To compare the model performance with different inputs, CT_in_ and CT_ex_ from the 4DCT were used as inputs to generate the corresponding ventilation images (CTVI_DLCT_). Moreover, the results of the network using the test dataset were compared with those of a radiomics‐based method (CTVI_FM_),^17^ a supervoxel‐based method (CTVI_SVD_),^18^ the Unet‐based method (CTVI_Unet_),^37^ and the two traditional DIR‐based methods CTVI_Jac_^13^ and CTVI_HU_.^14^ In CTVI_FM_, the time‐averaged 4DCT images were divided into patches, and labeled as defective or non‐defective regions. Relevant features were identified by extracting intensity and texture information, and feature maps (FMs) were generated using a voxel‐based radiomics approach. CTVI_SVD_ utilized simple linear iterative clustering^38^ to segment the lung volume supervoxels, which were then applied to calculate the average density value (D_mean_) for ventilation image generation. For a direct comparison, the standard Unet (CTVI_Unet_) architecture was used, featuring an analysis and synthesis path with four resolution steps, each containing two 3 × 3 × 3 convolutions followed by ReLU activation and 2 × 2 × 2 max pooling. The Bonferroni correction^39^ was used to account for multiple comparisons. Given that six methods were compared, resulting in a total of 15 pairwise comparisons, the significance level was adjusted to *p* = 0.0033 using the Bonferroni correction.

For voxel‐wise correlation comparisons, Spearman's correlation coefficient (*R*) was calculated for the entire lung region between CTVIs and RefVIs for each participant. *R* represents the degree of correlation between two distributions, ranging from −1 to +1, with −1 indicating a perfect negative correlation and +1 indicating a perfect positive correlation, as defined by Equation (2):

(2) $$R=\frac{\sum_{i=1}^{N}\left[\left(y_{i}-\bar{y}\right)\left(p_{i}-\bar{p}\right)\right]}{\sqrt{\sum_{i=1}^{N}{\left(y_{i}-\bar{y}\right)}^{2}}\sqrt{\sum_{i=1}^{N}{\left(p_{i}-\bar{p}\right)}^{2}}}\;\;$$

where $\bar{p}$, $\bar{y}$, $p_{i}$, and $y_{i}$ denote the average value and the value at voxel *i* for the predicted and ground truth images, respectively. *N* denotes the total number of non‐zero voxels.

For similarity comparisons, an image intensity threshold of 0.66 was used to separate the high‐functional lung regions from the low‐functional lung regions. Here DSC_h_ and DSC_l_ stand for Dice similarity coefficients of high‐functional region and low‐functional region, respectively. The similarity between RefVI and each CTVI was evaluated by calculating the DSC for each patient, as defined by Equation (3):

(3) $$\mathrm{DSC}\;\left(A,B\right)=\frac{2\left|A\cap B\right|}{\left|A\right|+\left|B\right|}\;$$

where *A* indicates functional lung volumes in RefVI and *B* indicates the same functional lung volumes in CTVIs.

### 2.6 Ablation study

To evaluate the role of the Jacobian map in the generation of the ventilation image, an ablation study was conducted using only the average CT image as the model input to generate the ventilation image (CTVI_DualCT_). Only one FEB was used to extract features from the average CT to feed the fusion layers. The model's performance was assessed using a threefold cross‐validation approach and compared with CTVI_Dual_ on the test dataset.

## 3 RESULTS

### 3.1 Threefold cross‐validation

Tables 2 and 3 summarize the results of the three‐fold cross‐validation for voxel‐wise *R*, DSC_h_, and DSC_l_ between CTVI_Dual_ and RefVI for the validation and test datasets. In the validation set, the method achieved average evaluation scores of 0.74 ± 0.10 for *R*, 0.66 ± 0.11 for DSC_h_, and 0.82 ± 0.06 for DSC_l_. When applied to the test set, these metrics slightly decreased to 0.70 ± 0.10 for *R*, 0.64 ± 0.09 for DSC_h_, and 0.80 ± 0.04 for DSC_l_, respectively. For the model trained without augmentation, *R*, DSC_h_, and DSC_l_ were respectively 0.66 ± 0.09, 0.63 ± 0.09, and 0.77 ± 0.05, in the test dataset. These values were 0.04, 0.01, and 0.03 lower than those obtained by the model trained with data augmentation.

**TABLE 2:** The prediction performance for the three‐fold cross‐validation on validation datasets.

		*R*	DSC_h_	DSC_l_	*p*‐value (*R*)
Fold 1	CTVI_Dual_	0.76 ± 0.06	0.68 ± 0.09	0.83 ± 0.05	0.10
	CTVI_DLCT_	0.73 ± 0.07	0.70 ± 0.06	0.84 ± 0.04	
Fold 2	CTVI_Dual_	0.73 ± 0.12	0.66 ± 0.09	0.82 ± 0.05	0.08
	CTVI_DLCT_	0.69 ± 0.13	0.65 ± 0.13	0.80 ± 0.05	
Fold 3	CTVI_Dual_	0.73 ± 0.11	0.65 ± 0.13	0.81 ± 0.06	0.18
	CTVI_DLCT_	0.70 ± 0.12	0.60 ± 0.18	0.80 ± 0.07	
Average	CTVI_Dual_	0.74 ± 0.10	0.66 ± 0.11	0.82 ± 0.06	0.02
	CTVI_DLCT_	0.71 ± 0.11	0.65 ± 0.14	0.81 ± 0.06	

*Note*: The value was presented with mean ± SD format in the table. The *p*‐value was used to evaluate the Spearman's correlation between the CTVI_Dual_ and CTVI_DLCT_.

Abbreviations: CTVI, computed tomography ventilation imaging, CTVI_DLCT_, CT‐based deep learning method; CTVI_Dual_, synthesizing ventilation images; DSC_h_, the Dice similarity coefficients of high‐functional region; DSC_l_, the Dice similarity coefficients of low‐functional region; *R*, the Spearman's correlation coefficients.

**TABLE 3:** The model performance on test datasets.

		*R*	DSC_h_	DSC_l_	*p*‐value (*R*)
Fold 1	CTVI_Dual_	0.72 ± 0.10	0.65 ± 0.09	0.80 ± 0.04	0.03
	CTVI_DLCT_	0.69 ± 0.13	0.65 ± 0.09	0.80 ± 0.05	
Fold 2	CTVI_Dual_	0.68 ± 0.10	0.64 ± 0.08	0.80 ± 0.04	0.27
	CTVI_DLCT_	0.67 ± 0.14	0.63 ± 0.10	0.79 ± 0.05	
Fold 3	CTVI_Dual_	0.71 ± 0.10	0.65 ± 0.09	0.78 ± 0.04	0.06
	CTVI_DLCT_	0.67 ± 0.15	0.64 ± 0.10	0.78 ± 0.05	
Average	CTVI_Dual_	0.70 ± 0.10	0.64 ± 0.09	0.80 ± 0.04	0.01
	CTVI_DLCT_	0.68 ± 0.14	0.64 ± 0.10	0.79 ± 0.05	

*Note*: The value was presented with mean ± SD format in the table. The *p*‐value was used to evaluate the Spearman's correlation between the CTVI_Dual_ and CTVI_DLCT_.

Abbreviations: CTVI, computed tomography ventilation imaging, CTVI_DLCT_, CT‐based deep learning method; CTVI_Dual_, synthesizing ventilation images; DSC_h_, the Dice similarity coefficients of high‐functional region; DSC_l_, the Dice similarity coefficients of low‐functional region; *R*, the Spearman's correlation coefficients.

### 3.2 Comparison between CTVI_Dual_ and CTVI_DLCT_

**FIGURE 2:**
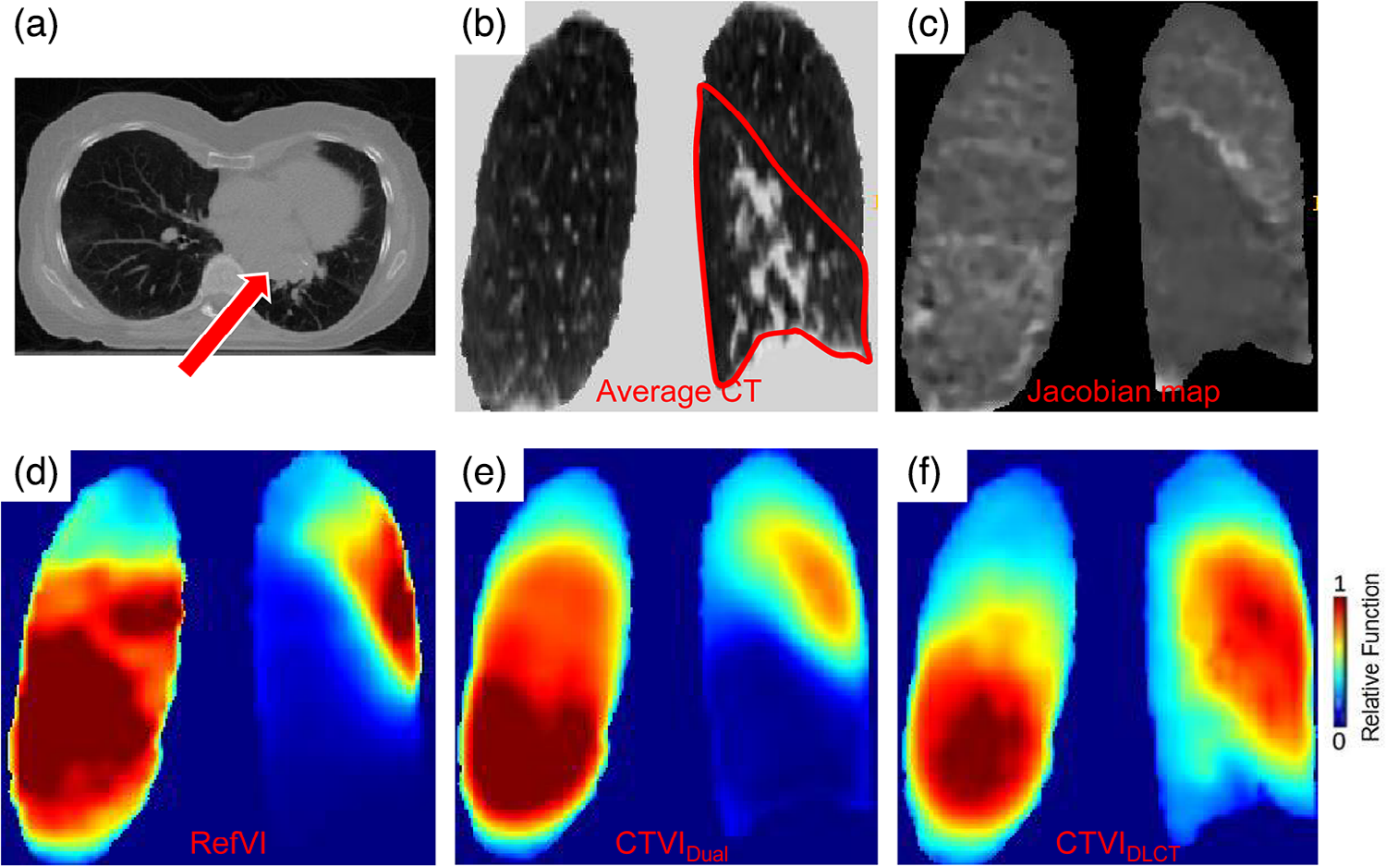
The comparison of the CTVI_Dual_ and CTVI_DLCT_ for a patient with tumor blocked the left lower lobe. (a) The tumor blocks the airway to left lower lobe as red arrow pointed in a transverse CT slice. (b and c) One coronal slice of the CT and Jacobian map of the input of CTVI_Dual_. The red contour in (b) is the lower lobe of the left lung. (d–f) The corresponding coronal slices of the RefVI, CTVI_Dual_, and CTVI_DLCT_. CTVI, computed tomography ventilation imaging, CTVI_DLCT_, CT‐based deep learning method; CTVI_Dual_, synthesizing ventilation images; RefVI, reference ventilation images.

The results of the comparisons between CTVI_Dual_ and CTVI_DLCT_ in different scenarios are shown in Tables 2 and 3. In the validation dataset, CTVI_DLCT_ achieved lower accuracy than CTVI_Dual_ in all evaluation indices, with differences of 0.03, 0.01, and 0.01 for *R*, DSC_h_, and DSC_l_, respectively. Similar results were observed in the test dataset, with CTVI_DLCT_ exhibiting lower accuracy than CTVI_Dual_ by 0.02 and 0.01 for *R* and DSC_l_, respectively. In both the validation and test datasets, the *p*‐values for *R* comparison were < 0.05, indicating a statistically significant difference. The results highlighted in Figure 2 demonstrate that CTVI_Dual_ can accurately identify and display low‐functional values in tumor‐blocked lung regions. This is particularly evident in Figure 2c, where the blocked regions of the lung show a consistent and homogeneously low value on the Jacobian map, indicating that these blocked regions experienced minimal volume changes during breathing. The use of the Jacobian map aids CTVI_Dual_ in recognizing blocked regions, as shown in Figure 2e. The DSC_l_ of CTVI_Dual_ was 0.01 higher than that of CTVI_DLCT_ in the validation and test datasets.

### 3.3 Comparison of CTVI_Dual_ with CTVI_FM_, CTVI_Unet_, CTVI_SVD_, CTVI_Jac_, and CTVI_HU_

**FIGURE 3:**
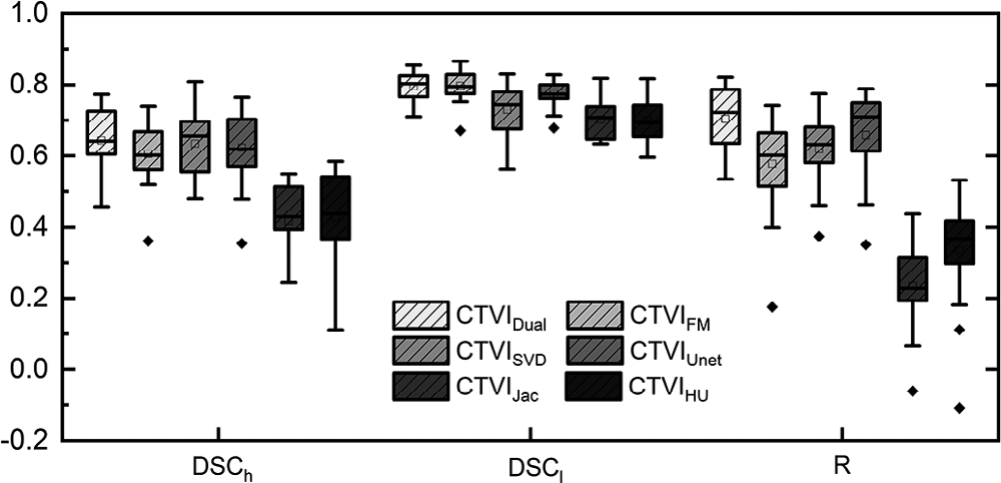
The comparison between the CTVI_Dual_, CTVI_FM_, CTVI_SVD_, CTVI_Unet_, CTVI_Jac_, and CTVI_HU_ on the test dataset. CTVI, computed tomography ventilation imaging; CTVI_Dual_, synthesizing ventilation images; CTVI_FM_, radiomics‐based method; CTVI_SVD_, super voxel‐based method; CTVI_Unet_, Unet‐based method.

Figure 3 compares the performance of CTVI_Dual_ with CTVI_FM,_ CTVI_Unet_, CTVI_SVD_, CTVI_Jac_, and CTVI_HU_ in the test dataset. In terms of *R*, CTVI_Dual_ showed a significantly higher *R* compared to CTVI_FM_, CTVI_Unet_, CTVI_SVD_, CTVI_Jac_, and CTVI_HU_, with differences of 0.12, 0.04, 0.08, 0.47, and 0.37, respectively. For CTVI_FM_, CTVI_Jac_, and CTVI_HU_, *R* was lower for CTVI_Dual_ with *p*‐values < 0.003. This indicates that CTVI_Dual_ was better correlated with RefVI than with other methods. For DSC_h_, CTVI_Dual_ had a higher value than CTVI_FM_, CTVI_Unet_, CTVI_Jac_, and CTVI_HU_, with differences of 0.03, 0.02, 0.23, and 0.22, respectively, comparable to that of CTVI_SVD_. However, only CTVI_Jac_ and CTVI_HU_ showed a significant difference of *p* < 0.003. Finally, for DSC_l_, CTVI_Dual_ had the same value as CTVI_FM_ but had a higher value than CTVI_SVD_, CTVI_Unet_, CTVI_Jac_, and CTVI_HU_, with differences of 0.07, 0.03, 0.10, and 0.10, respectively. The DSC_l_ values of CTVI_SVD_, CTVI_Jac_, and CTVI_HU_ were significantly lower than that of CTVI_Dual,_ with *p* < 0.003.

### 3.4 Results of the ablation study

CTVI_DualCT_ achieved a lower accuracy in all evaluation indices compared to CTVI_Dual_ in the test dataset, with differences of 4.6%, 0.6%, and 3.2% for *R*, DSC_h_, and DSC_l_, respectively. These results demonstrated that the Jacobian map provided complementary perspectives and information for the model.

### 3.5 Lung cancer patients with additional lung diseases

**FIGURE 4:**
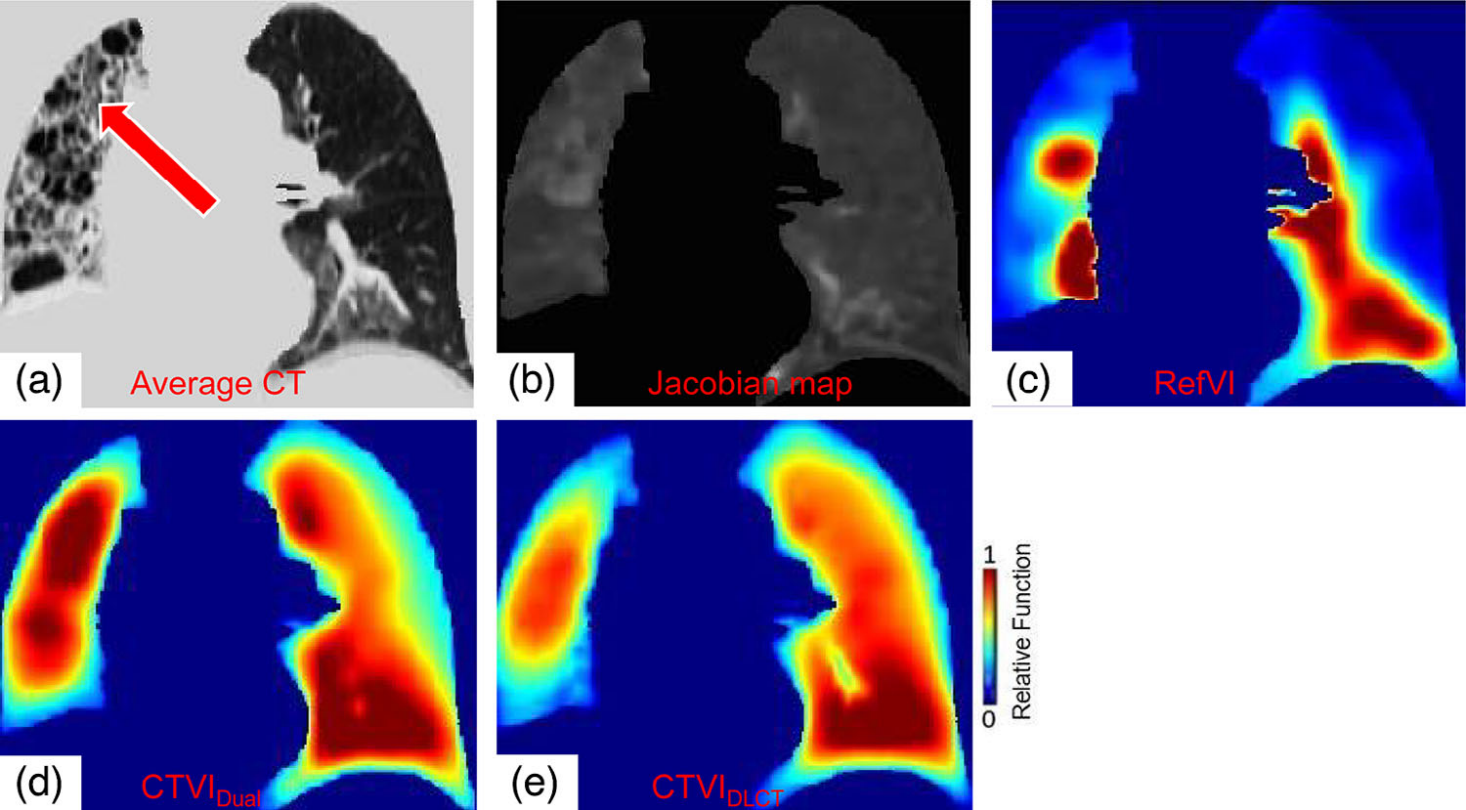
An example of patient with additional lung diseases. (a) One coronal slice of the CT image with additional lung diseases in the right lung as pointed by the red arrow. (b–e) The corresponding coronal slices of the Jacobian map, RefVI, CTVI_Dual_, and CTVI_DLCT_. CTVI, computed tomography ventilation imaging; CTVI_DLCT_, CT‐based deep learning method; CTVI_Dual_, synthesizing ventilation images; RefVI, reference ventilation images.

For lung cancer patients with additional lung diseases, CTVI_Dual_ yielded a low *R* with RefVI. As shown in Figure 4a, a defective lung region with heterogeneous density in the right lung caused a falsely high ventilation value Figure 4d. This disease feature was relatively rare and absent in the training dataset, and the Jacobian map showed heterogeneity in the right lung Figure 4b, resulting in the lowest *R* value for this patient (0.53).

## 4 DISCUSSION

This study introduces a novel CTVI approach that directly integrates both anatomical and motional information from 4DCT images. The proposed CTVI_Dual_ achieved a mean *R* of 0.70 (0.53–0.82) with the ground‐truth ventilation images, which was significantly higher than the correlations achieved by other methods: CTVI_FM_ (0.58, 0.18–0.74), CTVI_Jac_ (0.23, ‐0.06–0.43), and CTVI_HU_ (0.33, ‐0.11–0.53), all with *p* < 0.003. The DSC_h_ of CTVI_Dual_ was 0.64 (0.46–0.77), which was significantly higher than those of CTVI_Jac_ (0.42, 0.24–0.55, *p* < 0.003) and CTVI_HU_ (0.43, 0.11–0.58, *p* < 0.003). The DSC_l_ values of CTVI_SVD_ (0.73, 0.56–0.83), CTVI_Jac_ (0.70, 0.63–0.82), and CTVI_HU_ (0.70, 0.60–0.82) were lower than that of CTVI_Dual_ (0.80, 0.71–0.85), all with *p* < 0.003. In addition, CTVI_Dual_ outperformed CTVI_DLCT_, particularly in patients with obstructed lung regions. This indicates that incorporating the Jacobian map as input can guide the model in learning motional information and synthesizing more accurate ventilation images.

The use of the average CT image and the Jacobian map as inputs provided better performance compared to using CT_in_ and CT_ex_. Enhancements to CTVI_DLCT_ could be achieved by adding an extra module for direct learning of deformation information. For example, incorporating a convolutional block attention module (CBAM)^40^ learns the independent features, whereas the spatial attention module models the correlation between distinct spatial positions in the feature map, thereby improving the overall network performance. However, integrating a CBAM increases the computational cost during training and inference, as well as the storage requirements and model training time. Moreover, because a 3D model was used in this study, the introduction of additional modules may necessitate reducing the data input size, potentially decreasing the model's final accuracy.

The patient dataset in this study exhibited diverse lung ventilation patterns due to factors such as lung disease, tumor obstruction, and gravity effects. Figure 4 shows a weak correlation (*R* = 0.53) between CTVI_Dual_ and RefVI in patients with additional lung diseases. The heterogeneity of lung disease patterns can be challenging for deformable registration methods in accurately evaluating volume changes in diseased lung regions. The high signal in the right lung, as shown in Figure 4c, may have been caused by the deposition of the radioactive tracer at the hilus of the right lung. The *R* and DSC values varied among the three‐fold cross‐validations, indicating differing patient characteristics in each fold. Furthermore, the limited training dataset of 45 patients may have impacted the generalizability of the algorithms. Future studies should combine large and diverse datasets to verify model performance in different clinical applications.

The proposed network is generalizable to 4DCT datasets with different temporal resolutions. However, reduced temporal sampling limits temporal information. Averaging CT images can smooth temporal gaps and supplement missing information with the Jacobian matrix. Moreover, image quality issues, such as noise, reduced resolution, and artifacts, were common in these datasets. The average CT image generated by averaging multiple phases can effectively reduce noise and enhance image quality. Besides, a low temporal resolution can omit crucial moments in the respiratory cycle, thereby affecting the generation of ventilation images. By integrating CT images from multiple time points, the average CT image can restore lost information. The Jacobian matrix compensates for the lack of temporal information, thereby improving the integrity of the ventilation images. However, averaging CT images may blur fine structures. Future studies should incorporate image pyramids and super‐resolution reconstruction to further enhance details and clarity.

Yuan et al.,^23^ Meng et al.,^41^ and Kipritidis et al.^42^ have reported that lung function can change during radiation therapy. Our innovative model, which merges ventilation images, serves a dual purpose: it supports initial treatment planning and enables adaptive FLART. Adaptive re‐planning based on changes in lung function can help preserve lung function in patients after treatment. In addition, regions with low ventilation because of pressure from tumors in the central airway and blood vessels may recover after radiotherapy.^23^ Protecting these regions and minimizing the dose to normal lung regions is crucial. If tumor shrinkage occurs after treatment, patients may experience reduced dyspnea and increased lung function. Our model could identify blocked regions and assign them low‐functional values. Further investigation is needed to identify these regions and determine whether they can recover, thereby guiding initial treatment planning. Furthermore, an important challenge in applying CTVI_Dual_ to patients with blocked lung regions is determining whether the large defect regions resulted from tumor obstruction or are attributable to other pathological conditions. This differentiation is crucial and warrants further research to enhance diagnostic accuracy. Although the Jacobian map is generated through deformable registration, its accuracy can be compromised by the presence of artifacts. This limitation highlights the need for improved methodologies to mitigate the impact of artifacts on the registration process and ensure the reliability of the Jacobian map.

This research encountered several limitations, primarily associated with the model's reliance on Jacobian maps produced from deformable registration, which can be sensitive to artifacts present in 4DCT images.^43^ Enhancing deformable registration accuracy through artifact minimization in 4DCT images is critical for improvement. The RefVI used in this study contained artifacts such as hotspots located in the central airway caused by airway blockage. This could have misguided the model to generate false high‐functional regions. Future studies should utilize lung masks that exclude the airways and pulmonary vasculature to reduce such artifacts, thereby improving the accuracy of deformable registration.^31^, ^44^ In addition, further processing of 4DCT images using techniques such as image filtering, artifact removal algorithms, or image reconstruction may be used to reduce the artifacts. The utilization of more precise spatial interpolation methods can also enhance the accuracy of the generated Jacobian map by improving the interpolation of the 4DCT images. Moreover, considering the limitations imposed by hardware capabilities and the complexity of the model, the proposed network faces the challenge of training with a batch size of one. This design choice may increase the risk of overfitting during model training. To address this issue, data augmentation and regularization techniques should be utilized to improve the generalization. Furthermore, the adoption of transfer learning can help mitigate the drawbacks that affect network performance in constrained conditions. By leveraging a pretrained model as the initial parameter and fine‐tuning it with samples, the adverse effects on network performance can be mitigated within the given limitations.

The proposed network, despite its intricacy, presents substantial clinical benefits by integrating the average 4DCT with motional information derived from 4DCT. It is able to synthesize lung ventilation images. As the average 4DCT supplies crucial anatomical structure while the Jacobian map reveals dynamic lung volume shifts, a particularly valuable insight in cases with lung obstructions. Furthermore, the study's demonstration of cross‐modality generalization, training on PET images, and validating with SPECT, underscores its versatility. This capability is a significant step toward broad applicability. However, to further validate and enhance the clinical utility of this approach, it is imperative to expand the research with a larger, multi‐center patient dataset.

## 5 CONCLUSION

In this study, an anatomy and motion dual‐aware lung ventilation imaging method is developed to synthesize lung ventilation images using a dual‐aware‐based neural network. This approach demonstrates promising results, suggesting that deformation information can provide valuable supplementary inputs for estimating lung ventilation. Further research and development in this area are required to fully understand and utilize this approach in clinical applications.

## CONFLICT OF INTEREST STATEMENT

The authors declare no conflicts of interest.

## Supporting information



Supporting Information

Supporting Information

## Data Availability

Research data is not available at this time.
