# Supplementary figures and images for: Motion and anatomy dual aware lung ventilation imaging by integrating Jacobian map and average CT image using dual path fusion network
Source: Med Phys. 2024 Oct 21;52(1):246–56. doi: 10.1002/mp.17466 (PMC11700001; doi:10.1002/mp.17466)

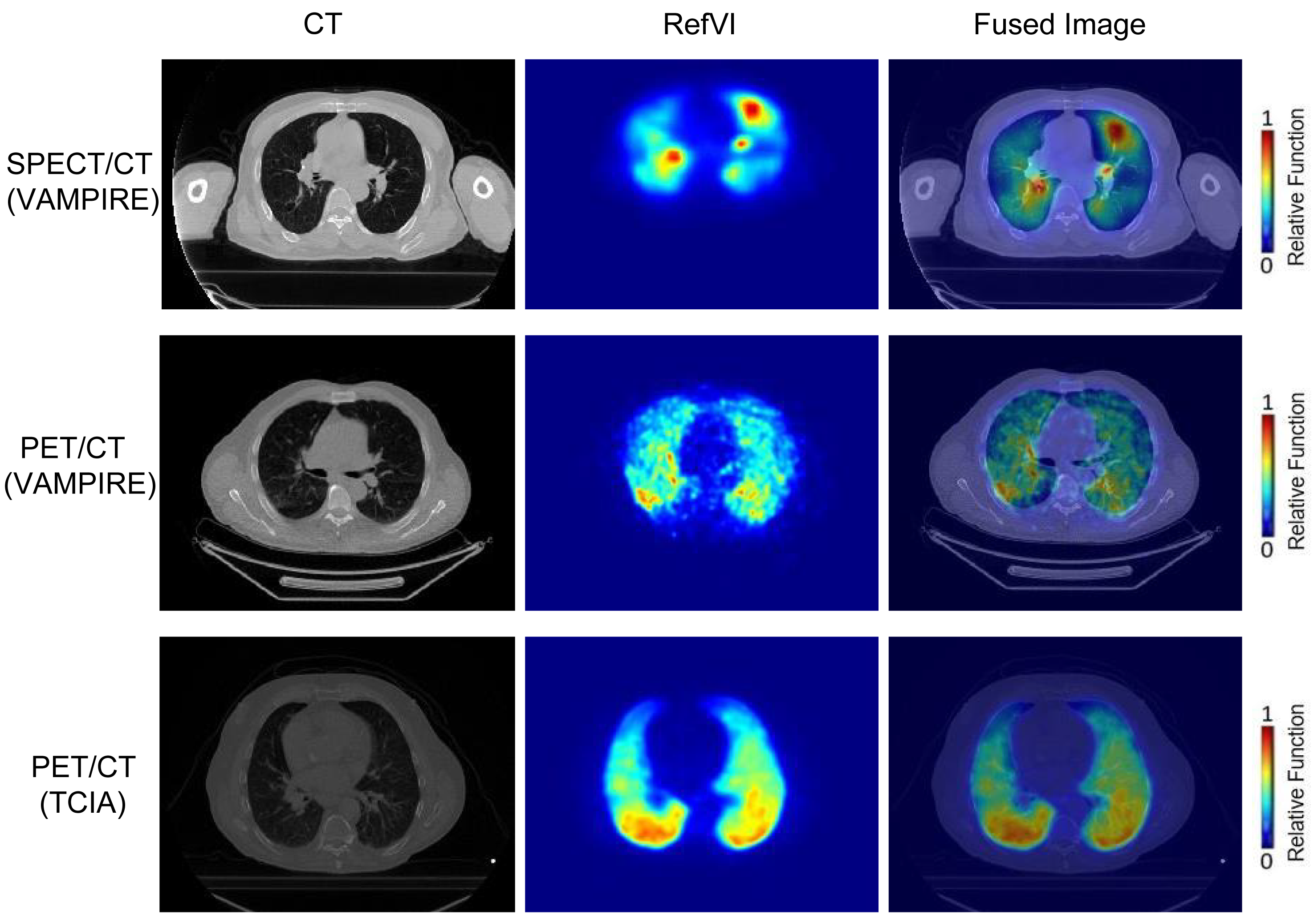

Supplement: Supplementary file 1 — Supporting Information [file MP-52-246-s001.tif]
