# Supplementary material for: Motion and anatomy dual aware lung ventilation imaging by integrating Jacobian map and average CT image using dual path fusion network
Source: Med Phys. 2024 Oct 21;52(1):246–56. doi: 10.1002/mp.17466 (PMC11700001; doi:10.1002/mp.17466)
Supplement: Supplementary file 2 — Supporting Information [file MP-52-246-s002.docx]

**Figure S-1.** Some selected patients’ images from the three datasets for demonstration. The fused Image is the overlap between the CT with RefVI.
